# Carpometacarpal Dislocation of the Third to Fifth Fingers and an Associated Fracture of the Hamate in a Military Paratrooper

**DOI:** 10.1155/2020/2861604

**Published:** 2020-06-30

**Authors:** Georgios Kalinterakis, Emmanouil Antonogiannakis, Arezoo Abdi, Georgios Demetriades, Alexandros Koulouktsis, Athanasios Syllaios, Antonios Koutras, Sotiria Vrouva, Anastasios Papagiavis, Miltiadis Ziogas

**Affiliations:** ^1^First Department of Orthopedics, 401 General Military Hospital of Athens, Athens 11525, Greece; ^2^First Department of Surgery, Laiko General Hospital, National and Kapodistrian University of Athens, Athens 11527, Greece

## Abstract

Multiple carpometacarpal dislocations with a simultaneous fracture of the hamate represent less than 1% of all injuries to the hand and wrist regions, with a scarcity of published cases. These injuries usually require a great force, and diagnosis can be missed or delayed because of the high likelihood of other severe concomitant injuries. We report a case of acute closed dislocation of the third through fifth carpometacarpal joints and an associated fracture of the hamate in a military paratrooper. The injury was caused by a wrong landing technique during parachuting. The patient was managed with primary surgical repair, and after a six-month follow-up, he has excellent functional results. The fact that both this clinical entity and the mechanism of injury are very unusual a high index of suspicion is needed, especially for orthopedic surgeons working in military hospitals. Additionally, given that there is a paucity of published cases and optional treatment is controversial, this study corroborates the superiority of surgical repair in a long-term basis.

## 1. Introduction

Carpometacarpal (CMC) dislocations of the hand with a simultaneous fracture of the hamate have been infrequently cited in the medical bibliography occurring in less than 1% of osseous hand injuries. The most common mechanism of the injury is the hyperflexion of the metacarpal heads. These injuries usually require a great force, and diagnosis can be missed or delayed because of the high likelihood of other severe concomitant injuries. Applying a high index of suspicion and performing a good clinical examination play a prominent role in recognizing this rare condition. Treatment is controversial and can be either conservative or operative [1–3]. We report a case of acute closed dislocation of the third through fifth carpometacarpal joints and an associated fracture of the hamate in a military paratrooper.

## 2. Case Presentation

A 22-year-old right-handed paratrooper was presented to the orthopedic emergency department because he fell on his outstretched right hand after having attempted a wrong landing technique. The accident took place in the island of Rodos where he received first aids and was then transported to our hospital for further treatment.

On physical examination, there was swelling and pain over the dorsum of the hand. Despite the swelling, it was feasible to notice deformity and to palpate the characteristic step-off created by the bases of the metacarpals. Active movement of the wrist and the fingers was limited while the patient was unwilling to cooperate due to pain. Neurovascular assessment revealed both a strong palpable radial pulse and a lively capillary refill while sensation of the hand was normal.

**Figure 1:**
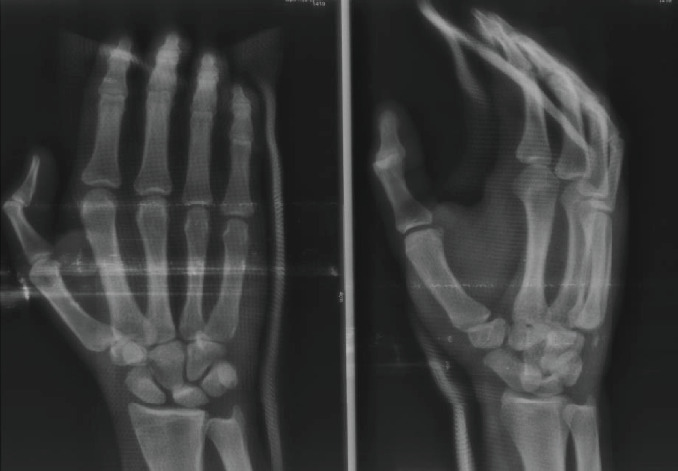
Anteroposterior and oblique radiographs of the right hand demonstrating complete dorsal dislocation of the three ulnar CMC joints.

**Figure 2:**
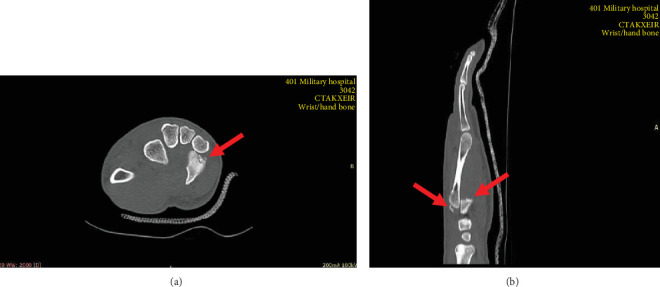
(a, b) CT images showing the dorsal dislocation of the fifth CMC joint and a bony fragment at the base of the fifth metacarpal that originated from the hamate (red arrows).

Plain radiographs (a dorsopalmar and an oblique view) of the right hand showed complete dorsal dislocation of the three ulnar CMC joints (Figure 1). In order to achieve a better assessment of the injured joint surfaces, an additional CT scan of the right hand was conducted. The latter not only confirmed the diagnosis but also revealed a bony fragment at the base of the fifth metacarpal that originated from the hamate (Figures 2(a) and 2(b)).

**Figure 3:**
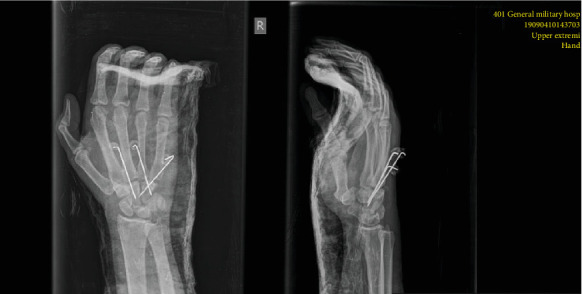
Radiographs after open reduction and fixation with Kirschner wires.

At the emergency department, closed reduction was attempted by applying longitudinal traction to the involved digits with pressure over the bases of the dislocated metacarpals. After that, the wrist was immobilized with a plaster. Three days later, the patient was operated under general anaesthesia in supine position. The surgical treatment chosen was open reduction and internal fixation (ORIF). Two longitudinal incisions were made over the second and fourth web spaces addressing adjacent respective joints. At this point, it is of note that when the subcutaneous hematoma was evacuated and the extensor tendons were retraced with the surrounding loose connective tissues, we noticed that our initial closed reduction which took place in the emergency room has been lost. Therefore, a new one was achieved under image intensifier guidance, and then, internal fixation was accomplished with K-wires 1.6. In detail, the third and fourth metacarpals were transfixed on the trapezoid and on the capitate, respectively. The fifth metacarpal was pinned similarly on the hamate (Figure 3). Regarding the fracture of the hamate, it was extra-articular and its reduction was secured by an interosseous suture vicryl 2-0. After the fracture alignment and joint reduction were evaluated under image intensifier, all wounds were meticulously irrigated and then closed in layers. Lastly, a sterile dressing and volar splint were applied for six weeks.

**Figure 4:**
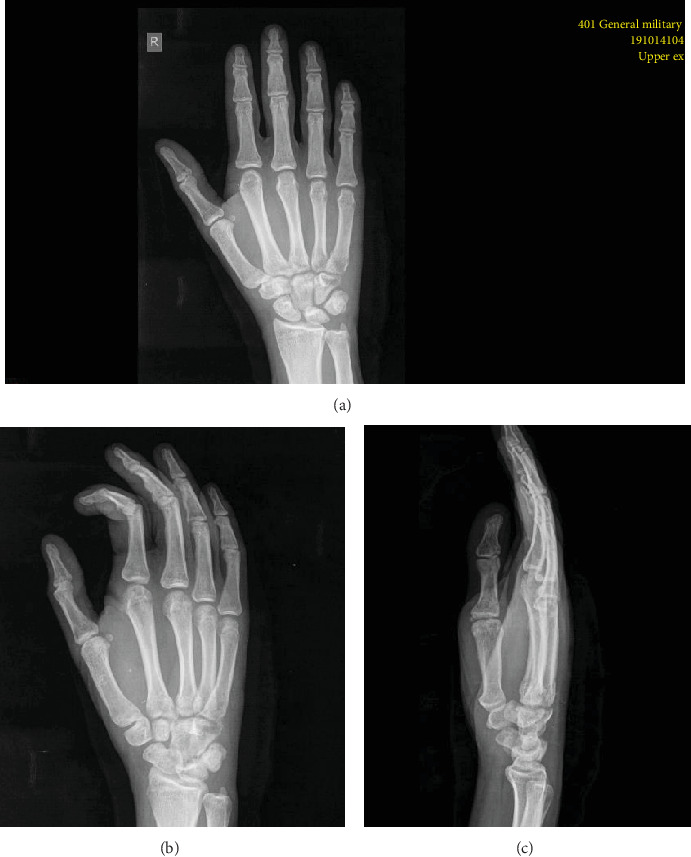
(a–c) Radiographs 6 weeks postoperatively after the K-wires' removal.

Both the splint and the K-wires were removed in six weeks' time (Figures 4(a)–4(c)). At this point, active ROM exercises were started. The DASH score one week after the splint removal was 62.5. At 8 weeks, physiotherapy was begun emphasizing on both strengthening exercises and earning full range of motion. In total, 10 sessions were done and the patient was reevaluated after one month. The DASH score was then 12.5. The patient was able to fully return to former activities in six months' time. The DASH score was 0.0. The last follow-up that took place 6 months after the injury revealed great functional results with full return of strength as well as satisfactory cosmetics results. The patient did not mention any difficulties in his labor and everyday life activities.

## 3. Discussion

Multiple carpometacarpal dislocations with a simultaneous fracture of the hamate are uncommon incidents, with a scarcity of published cases. They represent less than 1% of all injuries to the hand and wrist regions [3–6]. Additionally, owing to extensive swelling and overlapping of bones on the radiograph, up to 70% of them are missed or misdiagnosed [2, 7–9]. These injuries more commonly occur as a result of high-energy trauma as in our case (parachuting accident) while the predominant mechanism is direct force in the axial direction which causes secondary flexion or extension forces. The direction of CMC joint dislocation may be volar or dorsal depending on the direction of the force, with dorsal dislocations being the most common [5, 9, 10]. The CMC joint is the only joint not having a gliding configuration but instead is a modified saddle-shaped joint. Stability at the finger CMC joints is provided by static and dynamic constraints. The former refers to multiple articular facets while the latter to a system of four ligaments (dorsal, palmar, and 2 sets of interosseous) with surrounding muscles and tendons. What is more, it is essential to note that the third metacarpocapitate articulation is located more proximal than that of the other CMC joints, creating a so-called keystone relationship. As a result, reduction and stabilization of the third metacarpal CMC joint is fundamental for reduction of the remaining CMC joints. The same approach was also followed in our case. Furthermore, because of the tough attachments of the CMC ligaments, these injuries are often combined with avulsion fractures of the base of the metacarpal or the carpal bones, a finding noticeable in our case [3, 5, 6].

Dislocations of the CMC joint can be easily missed in the acute setting. Thus, it is recommended that three radiographs should be taken: a dorsopalmar, a true lateral, and an oblique view of the hand. However, a computed tomography (CT scan) is sometimes necessary in order to confirm the diagnosis and show missed carpal bone fractures, assisting in this way to preoperative planning [3, 11].

Because of the paucity of the published cases, treatment of carpometacarpal injuries is debatable. Two case reports by Horneff et al. and Agarwal A. and Agarwal R. demonstrated satisfactory results with closed reduction and splinting regarding the maintenance of reduction and the recovery of hand function [12, 13]. Operative treatment includes closed reduction and K-wire fixation or open reduction and internal fixation. The former is mostly chosen when either the joint surfaces are intact or in the absence of carpal fractures; otherwise, there is a high risk of redislocation. Fracture-dislocations often require open reduction and fixation (ORIF). This approach has been noted to have some distinct advantages as opposed to a closed reduction, including a more anatomical joint restoration, the drainage of local hematoma, and the prevention of tendon transfixation. The fixation can be achieved with K-wires or miniplates depending on the characteristics of the injury. In our case, due to the morphology of the injury, we used K-wires with excellent intra- and postoperative results [3, 5, 10, 11]. Finally, regardless of the treatment chosen, follow-up protocols suggest a period of immobilization, on average 4 to 6 weeks, at which point the splint and the pins (in case of surgical treatment) are removed, followed by hand and wrist physiotherapy so as to prevent postoperative stiffness and to improve the functional outcome.

Carpometacarpal (CMC) dislocations of the hand with a simultaneous fracture of the hamate comprise a rare injury. Despite the fact that the landing phase of a parachute jump mostly causes injuries in the lower extremities [14], upper extremities run also the risk of being injured. Thus, orthopedic surgeons must be aware of this clinical feature especially if they work in military hospitals. Given that there is a scarcity of published cases and optional treatment is controversial, this study corroborates the superiority of surgical repair in a long-term basis and acts as a contributory factor in medical literature in terms of guiding treatment.

## Data Availability

The data used to support the findings of this study are available from the corresponding author upon request.
